# Real-Time Simulation of Low-Energy Quantum Scattering with Quantum Circuits

**DOI:** 10.1021/acs.jpclett.6c02134

**Published:** 2026-09-08

**Authors:** Joshua M. Courtney, Phillip C. Stancil

**Affiliations:** † Department of Physics and Astronomy and the Center for Simulational Physics, 1355 University of Georgia, Athens, Georgia 30602, United States; ‡ Department of Physics and Astronomy, 1355 University of Georgia, Athens, Georgia 30602, United States

## Abstract

Simulating chemical dynamics such as atomic and molecular scattering is a natural target for quantum computation, where conventional (classical) hardware faces exponential cost. We present a product-formula quantum algorithm that models single-channel helium–helium scattering by real-time evolution of a Gaussian wavepacket, requiring no precomputation of eigenstates. Interaction with the Lennard-Jones potential and centrifugal barrier is applied unitarily with closed-form circuits, with the evolution processed directly with error-free quantum circuit compilation. Tracking the wave function results in converged phase shifts, from which the deflection function, scattering amplitude, and differential cross section are calculated. Identical-boson integral cross sections agree with time-independent Numerov and log-derivative benchmarks to 6.1%, with a localized residual in *s*-wave scattering. The construction realizes any potential of the form ∑_*i*_ *r* ^–*m*_*i*_^ with the same gate count as a single 1/*r* term, scales efficiently to multichannel dynamics, and provides a ground-truth operator for benchmarking approximate quantum simulation.

## Graphical abstract

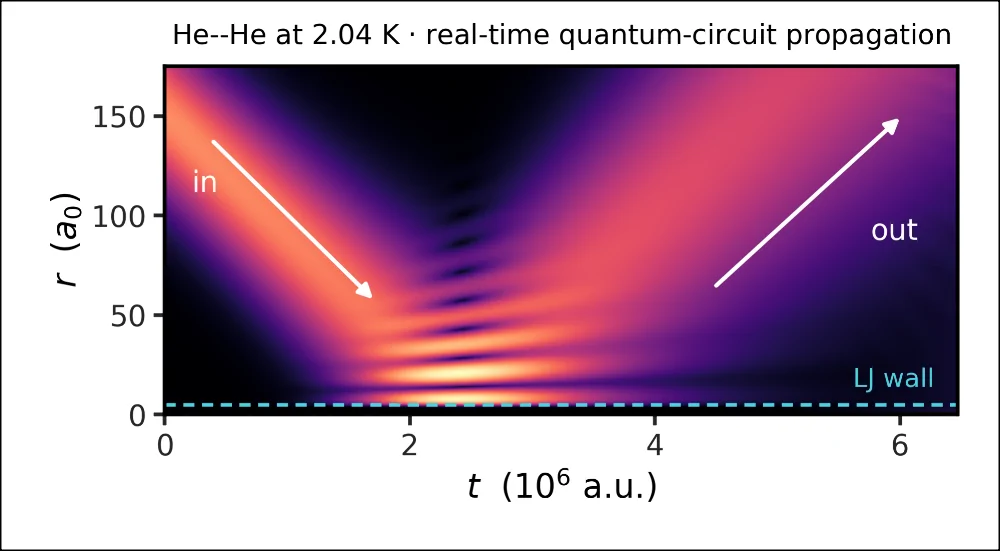

Atomic and molecular scattering underlies reaction rates, spectral intensities, and the comparison of theory with experiment across chemistry, astrophysics, and nuclear physics.^1,2^ Quantum scattering in systems with many degrees of freedom suffers the curse of dimensionality: the state space grows exponentially, making the computation of all eigenstates prohibitive. Subspace methods and semiclassical approximations isolate specific interactions,^3–5^ and conventional computing has advanced to high-dimensional simulation, incorporating neural-network potentials, physics-informed machine learning, and GPU-accelerated multiconfigurational methods.^6–12^ These methods, however, remain bounded by the computational complexity of scattering.^13^ Quantum computation is a natural route to many-body and multichannel interactions,^14,15^ though chemistry is known as a high-overhead application, demanding many logical qubits and near-fault-tolerant gate fidelities.^16–21^ Successes include variational eigensolvers, phase estimation, eigenstate preparation by rodeo algorithms, and small-scale nonadiabatic dynamics on analog simulators.^22–28^ Recent algorithms extract scattering phase shifts by first obtaining eigenstates on conventional hardware and then evolving in real time.^29–31^ While this importantly reduces measurement overhead, eigenstate precomputation remains exponentially complex and leaves chemical scattering open to future advances in quantum simulation.

Here we evolve a generic Gaussian wavepacket in real time under a Trotterized Hamiltonian with no eigenstate precomputation. The effective potential is composed of a 12–6 Lennard-Jones (LJ) surface plus a centrifugal term for partial-wave analysis, applied as a closed-form analytical gate composition on a binary-encoded position register. We evolve the elastic collision of two ground-state helium atoms on an error-free statevector emulator built with the Qiskit SDK, extract phase shifts by spatial Fourier transform with reference subtraction, and validate against time-independent solutions of the radial Schrödinger equation by the Numerov and log-derivative methods.^32,33^ The algorithm uses *O*(2^*n*^) gates without arithmetic registers and *O*(*n* ^2^) with quantum multipliers, extending to generic sums of exponentiated inverse-polynomials e^–*iV*(*r*)^ = exp­(−*i*∑_*j*_ *c*_*j*_ *r* ^–*m*_*j*_^), $m_{j}\in Z^{+}$ , on a binary basis. We refer to “classical hardware” and “conventional computation” to denote calculations performed on nonquantum computers, to distinguish circuit emulation as a technique to construct and validate quantum algorithms at the gate level. Requiring no oracle or polynomial approximation, the present construction supplies an exact “ground truth” for benchmarking approximate methods through Hilbert–Schmidt distance and entanglement fidelity, and extends to *M* coupled channels with a ⌈log_2_ *M*⌉-qubit ancilla register.^34–43^

We solve the radial, time-dependent Schrödinger equation

$$i\hbar \frac{\partial }{\partial t}\psi \left(r,t\right)=\left[T+V_{\mathrm{eff}}\right]\psi \left(r,t\right)$$ 1

by real-time propagation, imposing that the eigenstates are unknown as a requisite condition of the propagator unless there exists a previous subroutine that handles (classically intractable) eigenstate precomputation. The initial state is generically a Gaussian wavepacket:

$$\psi \left(r,t_{0}\right)={\left(\frac{1}{2\pi \delta^{2}}\right)}^{1/4}e^{-{\left(r-r_{0}\right)}^{2}/4\delta^{2}}$$ 2

a superposition of standing waves that can be prepared variationally (e.g., a single-Fermion unitary coupled-cluster (UCC) ansatz, *O*(*n* ^2^) gates) or, as here, through conventional (classical) initialization.^42–47^ Operating in a binary position basis *x* = Δ∑_*j* = 0_^*n*–1^2^*j*^ *q*_*j*_ with *N* = 2^*n*^ grid points in a box of size *L* and resolution Δ = *L*/*N*, the kinetic term is a degree-2 polynomial in momentum applied between centered quantum Fourier transforms (cQFT):

$$T=\exp(-i\frac{{\left(p-p_{0}\right)}^{2}\tau }{2\mu \hbar })=\exp(-i\frac{\tau }{2\mu \hbar }\left(p_{0}^{2}-2p_{0}\Delta_{p}\sum_{j}2^{j}q_{j}+\Delta_{p}^{2}\sum_{j}2^{2j}q_{j}+2\Delta_{p}^{2}\sum_{j<l}2^{j+l}q_{j}q_{l}\right))$$ 3

where μ is the reduced mass, τ the Trotter time step, *p*_0_ the initial relative momentum applied as a Galilean boost (Lorentz if relativistic), and Δ_*p*_ = 2*πℏ*/*L* the momentum grid spacing. The effective potential requires the central construction of this work.

We represent any inverse-power potential exactly on the qubit grid. The grid value of *x* is a global function of all basis states held in superposition, a conditional sum in a denominator is ill-defined for qubits. We impose the multilinear form

$$\frac{1}{x}=\underset{S\subseteq \left\{0,1,...,n-1\right\}}{\sum }a_{S}\underset{j\in S}{\prod }q_{j}$$ 4

whose coefficients follow exactly from a Möbius inversion

$$a_{S}=\underset{T\subseteq S}{\sum }{\left(-1\right)}^{\left|S\right|-\left|T\right|}\frac{1}{\Delta \underset{j\in T}{\sum }2^{j}},T\subseteq S\subseteq \left\{0,1,...,n-1\right\}$$ 5

adopting a convention where the sum over the empty index set is zero to avoid divergence at *V* (*x* = 0).

Products exponentiate to |*S*|-fold controlled phase rotations, so a 1/*r* potential is the circuit

$$U=\underset{S\subseteq \left\{0,1,...,n-1\right\}}{\prod }C^{\left|S\right|}R_{z}\left(2\pi \alpha a_{S}\right)$$ 6

A worked *n* = 2 example and the explicit algorithm are given in the Supporting Information (SI).

Higher inverse powers cost no extra gates. Squaring the circuit and using *q*_*j*_^2^ = *q*_*j*_ collapses 1/*x* ^2^. Inductively, any 1/*x* ^*m*^ maps onto the same set of subsets *T* by tensor product space commutativity, so coefficients are absorbed into the same |*T*|-fold controlled *R*_*z*_ gates as the base 1/*r* term (SI). Implementing 1/*r* ^*m*^ therefore requires exactly the same 2^*n*^ – 1 multicontrolled rotations as 1/*r*, and any *V*(*r*) = ∑_*i*_α_*i*_ *r* ^–*m*_*i*_^ is realized with no more gates than a single 1/*r* term. The centrifugal barrier for partial wave l , *V*_*cf*_ = $l\left(l+1\right)/\left(2\mu r^{2}\right)$ , is thus added at zero additional depth, and the 12–6 Lennard-Jones effective potential

$$V_{\mathrm{eff}}\left(r\right)=4\epsilon \left[{\left(\frac{\sigma }{r}\right)}^{12}-{\left(\frac{\sigma }{r}\right)}^{6}\right]+\frac{l\left(l+1\right)}{2\mu r^{2}}$$ 7

is assembled by summing the independently computed coefficients of each power into one circuit. These coefficients depend only on register size and inverse power. A library for *n* = 3–15 and *m* = 1–16 (Data Availability) was computed in under 100 core-hours and is fully parallelizable, with numerical precision a concern only for the smallest rotation angles (SI). The gate count scales as *O*(2^*n*^) before decomposition into two-qubit gates, reducing to *O*(2^*n*–1^) layers. Its principal advantage is multichannel scaling: *M* coupled electronic channels are handled by a ⌈log_2_ *M*⌉-qubit ancilla register that controls activation of the surfaces, adding only that factor to the exponent.^42,43^ Applying a Walsh–Hadamard transform to the multicontrolled gate composition results in a circuit for potential surfaces comprised of CNOT gates and uncontrolled *R*_*z*_ gates.^48^

Real-time evolution uses a second-order Trotter expansion:

$$U=e^{-iHt}\approx {\left[e^{-iT\tau /2}e^{-iV\tau }e^{-iT\tau /2}\right]}^{k}$$ 8

requiring only the kinetic term, the closed-form effective potential, and the initial wavepacket. The Trotter circuit and an expanded *n* = 4 potential block are shown in Figure 1a,b; Figure 1c,d illustrates the inverse-polynomial construction for a proton in a Coulomb potential.

**Figure 1:**
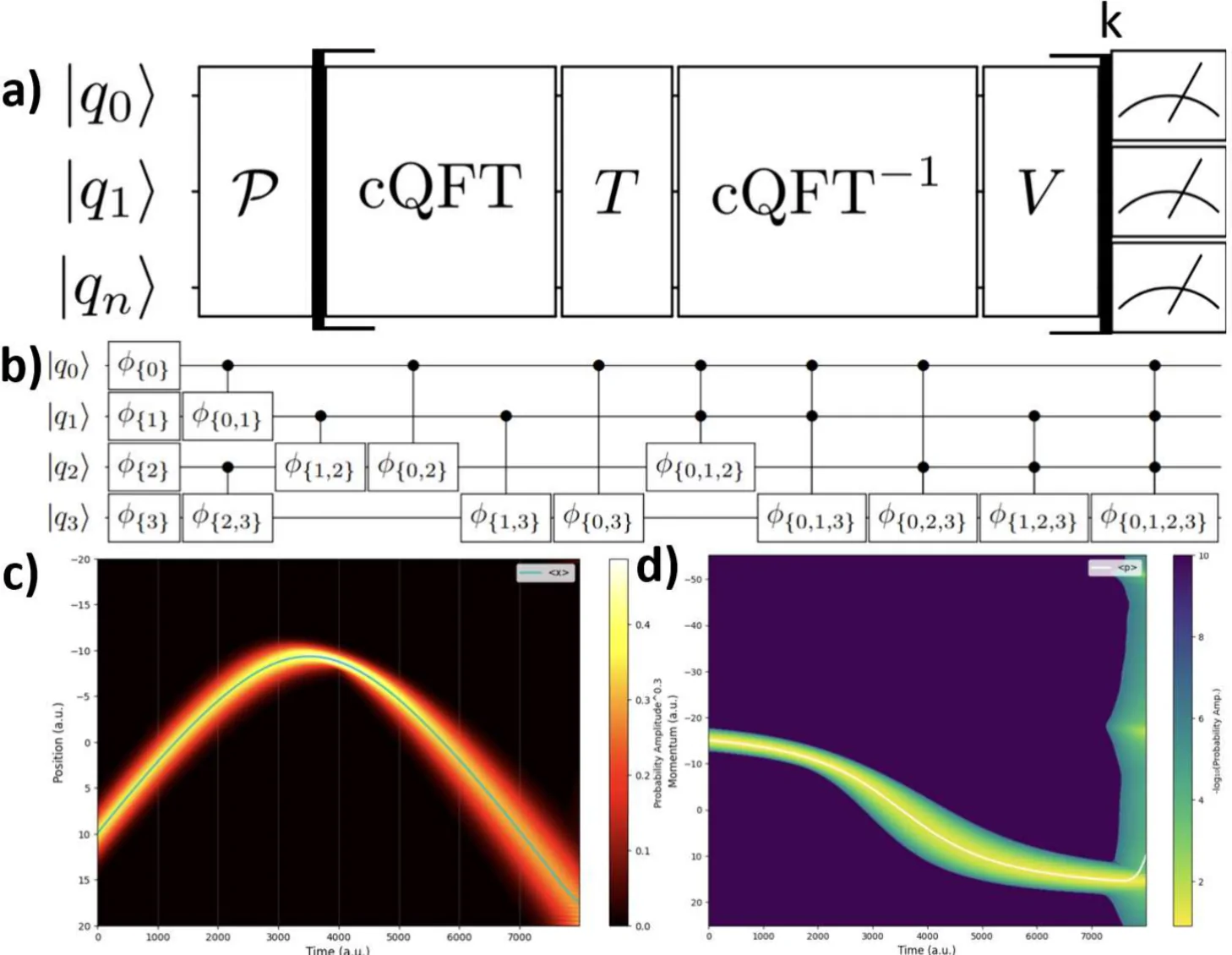
(a) Circuit diagram for the Trotterized Hamiltonian. After state preparation, the Trotter step is iterated over *k* time steps, and all qubits are measured. (b) *n* = 4-qubit circuit for an inverse-polynomial potential *V*(*r*) = 1/*r*. (c) Position heatmap for a proton in a Coulomb potential, initialized with *m* = 1836 a.u. at *x* = 10, *p*_0_ = −15, and δ = 1.0 in a box with *L* = 40 a.u. and *n* = 9 qubits; the circuit in (a) was applied for *k* = 800 time steps. Probability amplitudes are scaled by a power of 0.3 for visualization. Near *t* = 7500 a.u. the wavepacket begins to interact with the back of the potential through the periodic boundary intrinsic to the qubit basis. (d) Corresponding momentum heatmap to (c), obtained by measuring qubits after a centered QFT and scaled as −log­(|*ψ̃*|^2^).

To demonstrate a full scattering event we model the elastic collision of two ground-state He atoms in the 12–6 potential, evolving 19 partial waves (l=0 –18) with the full *V*_eff_ and a reference set with only *V*_*cf*_. Phase shifts are obtained from a spatial Fourier transform (FFT) of recorded wave function snapshots after the collision.^49^ The lab-frame momentum distribution *ψ̃*(*k*, *t*) = FFT­[ψ­(*r*, *t*)­e^*ik*_0_ *r*^] separates outgoing (*k* > 0) from incoming components, and for l>0 the interaction *S*-matrix follows as the ratio of outgoing amplitudes:

$$S_{\mathrm{LJ}}\left(l,k\right)=\frac{{\mathrm{\psi ̃}}_{\mathrm{full}}\left(k>0,t\right)}{{\mathrm{\psi ̃}}_{\mathrm{ref}}\left(k>0,t\right)}=e^{2i\delta_{\mathrm{LJ}}\left(l,E\right)}$$ 9

with *E* = *ℏ* ^2^ *k* ^2^/2μ; the *s*-wave case, where the barrier-free reference produces no outgoing wave, is treated through the known incoming amplitude (SI).^50^ From the converged phase shifts $\left\{\delta_{\mathrm{LJ}}\left(l,E\right)\right\}$ we form the scattering amplitude and the differential and total cross sections:^50^

f(θ,E)=12ik∑l=0lmax(2l+1)(e2iδl−1)Pl(cos⁡θ),dσdΩ=|f|2,σtot=4πk2∑l=0lmax(2l+1)sin2⁡δl 10

The phase shifts, the deflection function $\Theta \left(l\right)=2\left(\delta_{l}-\delta_{l+1}\right)$ , the scattering amplitude, and the differential cross section all agree with both time-independent benchmarks (Figure 2).

**Figure 2:**
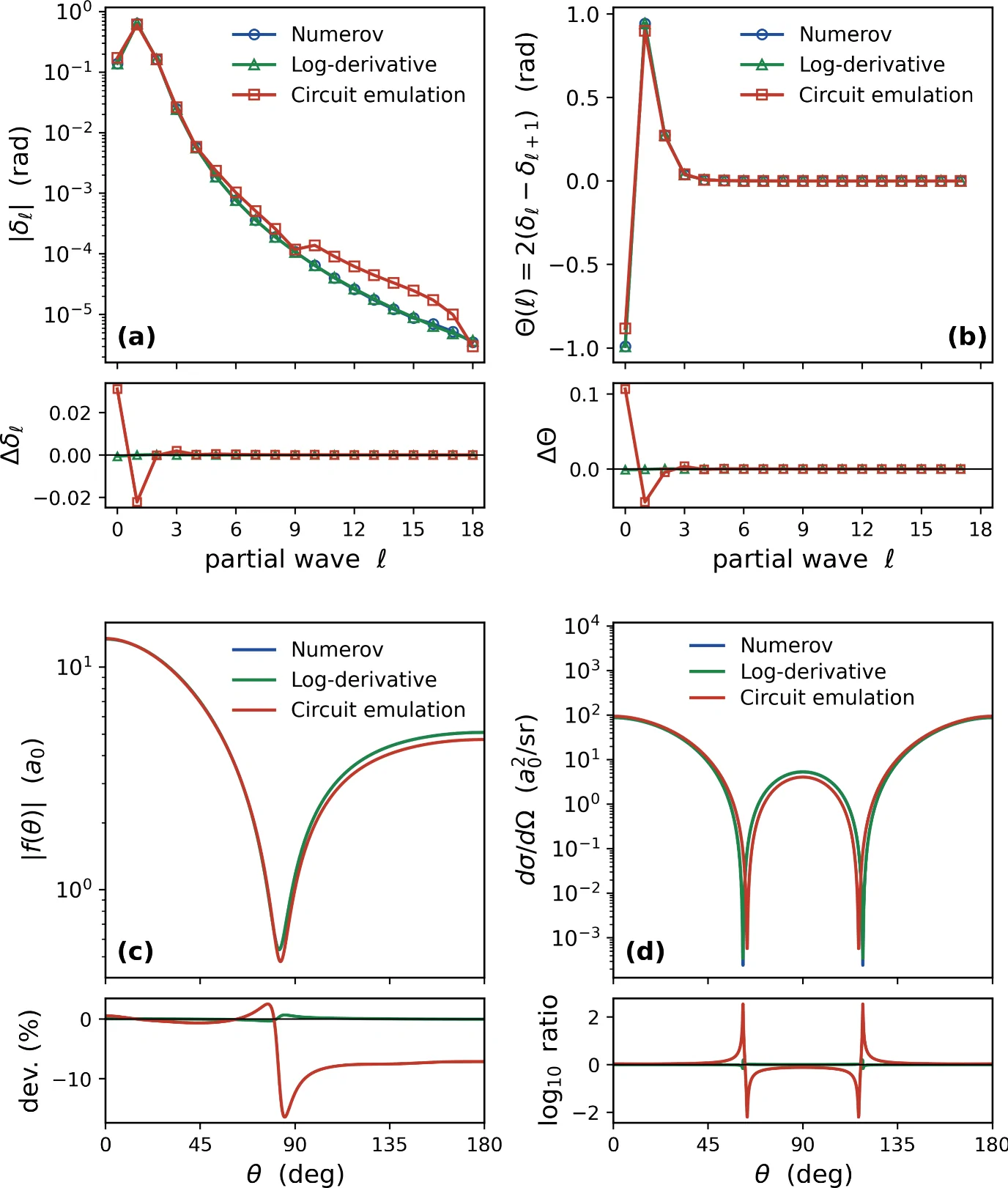
He–He elastic scattering at *E* = 2.043 K. Blue, green, and red denote Numerov, log-derivative, and quantum circuit emulation, respectively. (a) Phase shifts $\left|\delta_{l}\right|$ from the spatial FFT of the circuit-propagated wavepacket with residual $\Delta \delta_{l}$ . (b) Deflection function, tracking $\delta_{l}-\delta_{l+1}$ with residual ΔΘ. (c) Scattering amplitude |*f*(θ)| for distinguishable particles with residual given as a percentage. (d) Identical-boson differential cross section. Integral cross sections for both conventions (in/distinguishable) are given in the text. Residual is given as log_10_ of the ratio for clarity without diffraction nulls for small phase shifts.

After all calculations, we apply identical-particle symmetrization for ^4^He:

$$\sigma_{\mathrm{bos}}=\left(8\pi /k^{2}\right)\underset{l\;\mathrm{even}}{\sum }\left(2l+1\right)\;\sin^{2}\delta_{l}$$

Integral cross sections are calculated as 85.14 a_0_^2^ (circuit emulation), 80.28 a_0_^2^ (Numerov), and 80.39 a_0_^2^ (log-derivative), associated with distinguishable-particle values are 307.76, 321.91, and 321.94 a_0_^2^, respectively. Time-independent methods (as benchmarks) agree to 0.11% after symmetrization. The circuit result differs from Numerov by +6.05% in the symmetrized convention. We identify the residual as being mostly localized to *s*-wave scattering, seen when l=0 is set apart, after which the circuit and time-independent results agree to 0.28%.

This construction provides a closed-form quantum circuit for inverse-polynomial potentials on a binary basis, shifting exponential complexity to a one-time precomputation of rotation angles and to the *O*(2^*n*^) gate count, the latter of which can be made *O*(*n* ^2^) with quantum arithmetic, though resource calculations for optimal application become application-specific (SI).

The logical qubit count in the position register *n* scales with the characteristic energy of the system and the simulation box size as

$$n_{\min}=\left\lceil \log_{2}\left(\frac{L\sqrt{2\mu E_{\mathrm{char}}}}{\pi }\right)\right\rceil$$ 11

where *E*_char_ is defined by the Nyquist region defining momentum space bounds, in which the bulk of the dual wave function propagates.

Momentum resolution is the largest factor in the accuracy of an extractable phase shift. Since the *S*-matrix is read at $k_{0}=\sqrt{2\mu E}$ on a grid with spacing Δ_*p*_ = 2*πℏ*/*L*, resolution is given as

$$\frac{\Delta_{p}}{k_{0}}=\frac{2\pi }{L\sqrt{2\mu E}}$$ 12

A resolution *ε*_*k*_ requires *L* ≥ 2π/(*ε*_*k*_ *k*_0_), with phase shift uncertainty bounded by

$$\varepsilon_{\delta }≲\left|\frac{d\delta_{l}}{dk}\right|\frac{\pi }{L}$$ 13

the worst case corresponding to a half-grid offset. The kinetic term is applied between centered QFTs as a diagonal operator, so the register must represent the largest non-negligible spectral value reached in the classical mechanically forbidden region $k_{\mathrm{wall}}=\sqrt{2\mu V_{\mathrm{cut}}}$ , where the WKB amplitude tolerance determines the cutoff potential height *V*_cut_. This condition is prevalent in first-quantized simulation and not specialized to quantum computing, set by Nyquist limits as 2^*n*^ ≥ *Lk*_wall_/π, equivalent to eq 11, recast as

$$n_{\min}=\left\lceil \log_{2}\left(\frac{L\sqrt{2\mu V_{\mathrm{cut}}}}{\pi }\right)\right\rceil$$ 14

Resolution sets a floor on *L*, and Nyquist limits demand a ceiling on *L*/2^*n*^, leaving a minimum position register size of

$$n_{\min}=\left\lceil \log_{2}\left(\frac{2}{\varepsilon_{k}}\sqrt{\frac{V_{\mathrm{cut}}}{E}}\right)\right\rceil$$ 15

Directly following, any first-quantized quantum simulation of a similar system that does not accommodate these limits will result in subconvergent observables. We specify the He–He application for proof-of-principle, where *V*_cut_ = 2.27 E_h_ and *ε*_*k*_ = 0.145 give *n*_min_ = 13.0. Higher collision energies relax the box requirement as *E* ^–1/2^, and raise the momentum content the grid must carry.

We find here that the above relations account *s*-wave residuals. Time-independent solutions provide $d\delta_{l}/dk$ for l= 0–3 as −5.74, +0.13, +2.41, and +0.45 *a*_0_. *s*-wave phase shifts are most sensitive to Δ_*p*_, varying approximately 40 times faster than *p*-wave shifts with wavenumber. With a working box size *L* = 200 *a*_0_, eq 13 bounds *s*-wave error at 0.090 rad, where the observed deviation relative to the benchmarks (0.031 rad) comfortably lives. Correction for *s*-wave scattering is a larger box, as error falls as 1/*L*.

Quantum signal processing offers optimal query complexity and maps the operator to a polynomial approximation.^34,51^ While preferred for the single-channel state, its query count grows with the Hamiltonian spectral norm, growing unfavorable for the strongly coupled, multichannel dynamics typical of chemical scattering and collision systems. Trotterization handles intersection seams invariantly, with commutator norms decreasing toward zero as diabatic surfaces cross.^52^ Moreover, a general potential energy surface (PES) is nonsparse, requiring *O*(2^*n*^) terms to qubitize the walk operator and favoring direct encoding on the position register. Thus, Trotterization remains valuable in quantum simulation despite suboptimal error scaling.^53^

An exact operator enables rigorous, hardware-agnostic benchmarking of approximate algorithms. We confirm in the SI that the Hilbert–Schmidt distance between this binary circuit and an independently derived binary-reflected Gray-code (BRGC) construction vanishes to numerical precision.^41^ Since the BRGC-encoded PES is provably efficiently approximable and the binary-to-Gray reflection is *O*(*n*), the binary encoding is efficiently approximable as well, though a concrete binary-basis approximation remains for future work. A validated ground truth permits variational compression, opening the work to variational fast-forwarding (for amenable systems), quantum-assisted compiling, and reinforcement-learning circuit synthesis, optimizing against operator-level metrics and a distributed T-budget instead of another approximation.^38,54–60^ Such approximations may be more favorable at higher energy and l as the scattering region moves away from the origin (SI).^34,35^

The scheme prioritizes preserving the structure of the wave function over storing eigenstates. In the multichannel extension, each of *M* surfaces contributes coefficients assembled into uniformly controlled rotations gated by a ⌈log_2_ *M*⌉-qubit channel register, raising the per-site cost from one *R*_*z*_ to *M* rotations interleaved with *M* – 1 CNOTs while preserving gate-count invariance under polynomial power.^42,43^

In summary, we have presented a real-time quantum-circuit emulation of an atomic scattering event with no precomputation of eigenstates. Phase shifts extracted by spatial FFT with reference subtraction reproduce the deflection function, scattering amplitude, and differential and integral cross sections of He–He scattering in agreement with independent Numerov and log-derivative calculations obtained on conventional hardware, and the Hilbert–Schmidt distance to the BRGC construction vanishes to numerical precision, giving operator-level validation without approximation. Practical deployment is currently limited by rotation-angle precision far below present gate fidelities and by the long evolution times required for phase-shift extraction,^18–21,29^ but the closed form bounds these costs and charts a path to efficient approximation. With the multichannel extension, this framework moves toward quantum simulation of coupled potential surfaces beyond the reach of conventional computational methods.

## Computational Methods

We propagate the 2^*n*^-amplitude statevector (grid-discretized wave function) using a compiled quantum circuit, tracking its evolution by recording the statevector at each time step without injecting shot noise, qubit decoherence, or gate operation error. The evolution is therefore representative of a “perfect” or “ideal” fault-tolerant quantum computer, with rotation angles limited only by floating-point precision. We define this as an “error-free statevector emulation” to distinguish the performed quantum gate operations from typical time-dependent conventional simulation. A Python script using the Qiskit-SDK, with C-integrated numerical preprocessing compiles a circuit whose gate-level logic is congruent to the script-level implementation.

A register of *n* = 13 qubits defines a position grid of resolution *L*/2^*n*^ ≈ 2.44 × 10^–2^ a.u. in a box of size *L* = 200 a.u. The wavepacket is initialized with width δ = 5.0 a.u., position *r*_0_ = 100 a.u., and momentum *p*_0_ = −0.2173 a.u., and propagated for *t* = 5 × 10^6^ a.u. with time step τ = 1.0 a.u. using the second-order Trotter expansion. This corresponds to a collision energy *E* = *ℏ* ^2^ *p*_0_^2^/2μ = 6.47 × 10^–6^ E_h_ ≈ 2.043 K. We begin phase-shift extraction at 1.1 *t*_scatter_ with *t*_scatter_ = *r*_0_μ/|*p*_0_| = 1.68 × 10^6^ a.u. the time required for the wavepacket to propagate to the origin.

The large box prevents wrap-around from the periodic boundary intrinsic to the qubit basis; absorbing boundaries are not straightforwardly unitary,^61,62^ so observables are extracted before high-momentum components reflect, a limitation that future work may address through Schrödingerization of non-Hermitian Hamiltonians, moment-matching dilations, or interleaved noncommuting walk operators.^62–64^ The Lennard-Jones parameters approximate fluid He at 2.044 K: ϵ = 3.236 × 10^–5^ E_h_, σ = 4.831 a.u., and *m*_He_ ≈ 7296.3 a.u., giving a reduced mass μ = *m*_He_/2.^65^ These parameters are chosen according to the elastic scattering model for fluid He at 2.044 K. Two sets of 19 partial-wave simulations (l=0 –18) were run, one with the full *V*_eff_ and a reference with only *V*_*cf*_.

Phase shifts were averaged over 500 sequential time steps after the separation of the outgoing wave. Uncertainty in calculated phase shifts from the time-dependent evolution arises from the scatter across time steps, which we provide alongside systematic error (eq 13).

Time-independent benchmarks used the Numerov^32^ and log-derivative^33^ methods. These benchmarks are executed on conventional hardware. The Numerov method uses three-point recursion to propagate the radial wave function. Matching to free-particle solutions in the asymptotic region recovers phase shifts. The log-derivative method propagates *Y* = *ψ′ψ* ^–1^, remaining numerically stable through classically forbidden regions. We give the system schematic in Figure 3.

**Figure 3:**
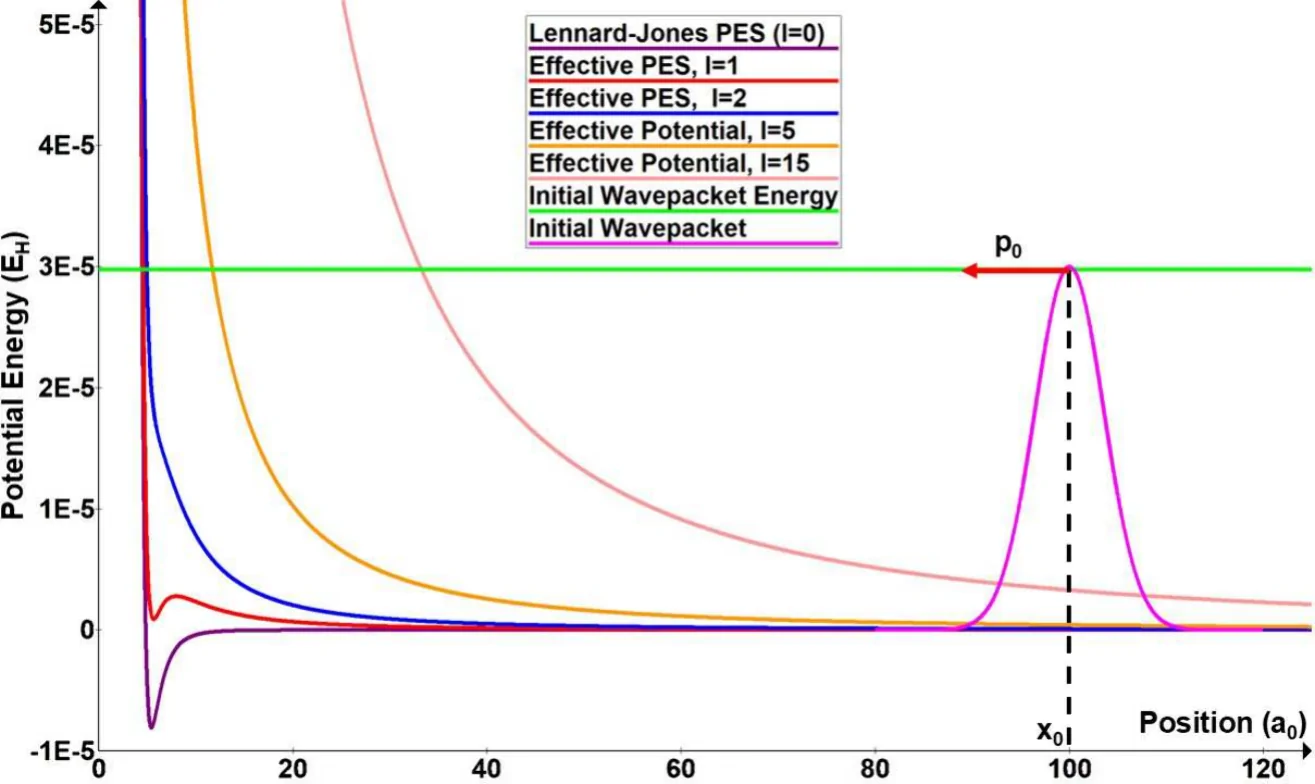
Schematic of the quantum scattering simulation by error-free statevector emulation. A Gaussian wavepacket is initialized at *x*_0_ = 100 a.u., allowing preparation by the variational quantum eigensolver (VQE) or direct (classical) optimization.^23,47^ The effective potential *V*_*LJ*_(*r*) + *V*_*cf*_(*r*) is shown with its components. The Hamiltonian is split so that the kinetic term applies the momentum as (*p* – *p*_0_)^2^/2μ and the potential term applies the full effective potential in a single circuit.

## Supplementary Material



## Data Availability

The Python (Qiskit SDK) scripts, the supporting C routines for numerical preprocessing, the precomputed coefficient library, and sample animations are available at this GitHub repository.

## Notes

Derivation of the multilinear potential coefficients with a worked *n* = 2 example; the repeated-squaring algorithm, coefficient recurrence, and complexity analysis; numerical precision of rotation angles; effective rotation-angle assembly; *s*-wave phase-shift extraction; the multichannel extension; operator-level validation via the Hilbert–Schmidt distance against the BRGC construction; and a quantum-arithmetic resource comparison, partial-wave truncation, momentum-resolution bound, and empty-sum convention (PDF)
