# Supplementary material for: Real-Time Simulation of Low-Energy Quantum Scattering with Quantum Circuits
Source: J Phys Chem Lett. 2026 Sep 8;17(38):10952–8. doi: 10.1021/acs.jpclett.6c02134 (PMC13618049; doi:10.1021/acs.jpclett.6c02134)
Supplement: Supplementary file 1 [file jz6c02134_si_001.pdf]

Supporting Information: Real-Time Simulation of Low-Energy Quantum Scattering with Quantum Circuits

Joshua M. Courtney* and Phillip C. Stancil

Department of Physics and Astronomy and the Center for Simulational Physics, University of Georgia, Athens, Georgia 30602, United States

E-mail: Joshua.Courtney1@uga.edu

Explicit Algorithm

The following section details how to construct potential energy surfaces from inverse polynomial functions using quantum circuits. Starting with the binary expansion for a number x

$$x = \sum_{j=0}^{n-1} q_j 2^j,\ q_j \in \{0, 1\} \tag{S1}$$

The primary stumbling block is from the direct substitution of this expression into $f(x) = 1/x$

$$f(x) = \frac{1}{x} = \frac{1}{\sum_{j=0}^{n-1} q_j 2^j} \tag{S2}$$

where realistically, the quantum state q_j in the denominator makes little physical sense. Instead, we assert the form

$$\frac{1}{x} = \sum_{S\subseteq\{0,1,\ldots,n-1\}} a_S \prod_{j\in S} q_j \tag{S3}$$

This equality states that all combinations of qubits q_j can express the function $1/x$ in a discretized basis, weighted by coefficients a_S for each subset of qubits. These coefficients are given by a Möbius inversion

$$a_S = \sum_{T\subseteq S} (-1)^{|S|-|T|} \cdot \frac{1}{\sum_{j\in T} 2^j} \tag{S4}$$

$|S|$ represents the cardinality of S, where by extension, $\sum_{j\in\varnothing} 2^j = 0$.

These products are exponentiated as phase rotations of a unitary $U = e^{2\pi i\phi(x)}$, where $\phi(x) = \alpha\frac{1}{x}$. For simplicity, we set $\alpha = 1$ as a scale factor. Broadly, α can be factored out and multiplied into base coefficients after the gate set has been established. This can be equivalently represented as controlled R_z rotations

$$U = \prod_{S\subseteq\{0,1,\ldots,n-1\}} C^{|S|} R_z(2\pi\alpha a_S) \tag{S5}$$

This becomes a product of $|S|$-fold controlled R_z gates acting on qubits in S.

For $n = 2$ qubits, we find the following coefficients:

$$
\begin{aligned}
a_{\varnothing} &= 0 \quad \text{(empty-sum convention; see below)} \\
a_{\{0\}} &= (-1)^{1-0} \cdot 0 + (-1)^{1-1} \cdot \frac{1}{2^0} = 1 \\
a_{\{1\}} &= (-1)^{1-0} \cdot 0 + (-1)^{1-1} \cdot \frac{1}{2^1} = \frac{1}{2} \\
a_{\{0,1\}} &= (-1)^{2-0} \cdot 0 + (-1)^{2-1} \cdot \frac{1}{2^0} + (-1)^{2-1} \cdot \frac{1}{2^1} + (-1)^{2-2} \cdot \frac{1}{2^0 + 2^1} = -\frac{7}{6}
\end{aligned} \tag{S6}
$$

The multilinear term is undefined for $T = \varnothing$ with a vanishing sum over the index set, so $\sum_{j \in \varnothing} 2^j = 0$. We set $a_{\varnothing} = 0$ accordingly (convention) instead of the continuum potential's $V(x = 0) = \infty$. This choice is largely without consequence, as the classical turning point at $E = 2.043\,\mathrm{K}$ is $r_t = 4.79\,a_0$ with a WKB adjustment of $\exp(-8.2) \approx 3 \times 10^{-4}$ at $r = 3.0\,a_0$ and $\exp(-52.6) \approx 10^{-23}$ at $r = 2.14\,a_0$. The error is small and reserved to low ℓ and higher E than what we presently consider.

This makes the unitary $U = e^{-\frac{i}{\hbar}V(x)}$, $V(x) = \frac{1}{x}$

$$
U = e^{-\frac{i}{\hbar}V(x)} = e^{-\frac{i}{\hbar}\sum_{S \subseteq \{0,1,\ldots,n-1\}} \left(\sum_{T \subseteq S} (-1)^{|S|-|T|} \cdot \frac{1}{\sum_{j \in T} 2^j} \right) \prod_{j \in S} q_j} \tag{S7}
$$

The coefficients a_S can be computed combinatorially, with the number of gates scaling exponentially with register size. Depth compression may allow for $\lfloor n/k \rfloor$ k-qubit operations to be performed in parallel,

Centrifugal Potentials

We have the following expression for $V(x) = \frac{1}{x}$, as

$$
V(x) = \frac{1}{x} = \sum_{S \subseteq \{0,1,\ldots,n-1\}} \left(\sum_{T \subseteq S} (-1)^{|S|-|T|} \cdot \frac{1}{\sum_{j \in T} 2^j} \right) \prod_{j \in S} q_j \tag{S8}
$$

A Lennard-Jones potential takes the form

$$V(x) = 4\epsilon\left(\left(\frac{\sigma}{r}\right)^{12} - \left(\frac{\sigma}{r}\right)^{6}\right) \tag{S9}$$

If we square our expression for $\frac{1}{x}$, we get

$$\begin{aligned}\frac{1}{x}\cdot\frac{1}{x} = \frac{1}{x^2} = &\left(\sum_{S\subseteq\{0,1,\dots,n-1\}}\left(\sum_{T\subseteq S}(-1)^{|S|-|T|}\cdot\frac{1}{\sum_{j\in T}2^j}\right)\prod_{j\in S}q_j\right)\\ &\times\left(\sum_{S_2\subseteq\{0,1,\dots,n-1\}}\left(\sum_{T_2\subseteq S_2}(-1)^{|S_2|-|T_2|}\cdot\frac{1}{\sum_{j\in T_2}2^j}\right)\prod_{j\in S_2}q_j\right)\end{aligned} \tag{S10}$$

$$\left(\frac{1}{x}\right)^2 = \sum_{S_1\subseteq\{0,1,\dots,n-1\}}\sum_{S_2\subseteq\{0,1,\dots,n-1\}} a_{S_1}a_{S_2}\prod_{j\in S_1}q_j\prod_{j\in S_2}q_j \tag{S11}$$

Since $q_j \in \{0,1\}$, we have $q_j^2 = q_j$ (idempotency of the Galois field $GF(2)$), so:

$$\prod_{j\in S_1}q_j\prod_{j\in S_2}q_j = \prod_{j\in S_1\cup S_2}q_j \tag{S12}$$

Grouping terms by their union:

$$\left(\frac{1}{x}\right)^2 = \sum_{T\subseteq\{0,1,\dots,n-1\}} b_T^{(2)}\prod_{j\in T}q_j \tag{S13}$$

where:

$$b_T^{(2)} = \sum_{\substack{S_1,S_2\subseteq\{0,1,\dots,n-1\}\\ S_1\cup S_2=T}} a_{S_1}\cdot a_{S_2} \tag{S14}$$

coefficients $a_S, a_{S'}$ are absorbed into the same $|S|$-fold controlled R_z gate when $S = S'$. So, an x^{-2} function does not require any more gates than x^{-1}.

We can therefore construct a unitary for the potential $V_{Centrifugal} = \frac{\ell(\ell+1)}{2\mu r^2}$ as

$$U = e^{-i\tau\ell(\ell+1)/(2\mu r^2)} = e^{-i\tau(\ell(\ell+1)/(2\mu))\sum_{T\subseteq\{0,1,\dots,n-1\}}\sum_{\substack{S_1,S_2\subseteq\{0,1,\dots,n-1\}\\ S_1\cup S_2=T}} a_{S_1}\cdot a_{S_2}\prod_{j\in T}q_j} \tag{S15}$$

Lennard-Jones in Quantum Simulation

By induction, for any positive integer k:

$$\left(\frac{1}{x}\right)^k = \sum_{T \subseteq \{0,1,\ldots,n-1\}} b_T^{(k)} \prod_{j \in T} q_j \tag{S16}$$

where:

$$b_T^{(k)} = \sum_{\substack{S_1, S_2, \ldots, S_k \subseteq \{0,1,\ldots,n-1\} \\ S_1 \cup S_2 \cup \ldots \cup S_k = T}} a_{S_1} \cdot a_{S_2} \cdot \ldots \cdot a_{S_k} \tag{S17}$$

Repeated Squaring Algorithm

For efficiency, we decompose degree k into powers of 2:

$$k = \sum_{i=0}^{\lfloor \log_2 k \rfloor} b_i 2^i, \quad b_i \in \{0, 1\} \tag{S18}$$

For example, $12 = 8 + 4 = 2^3 + 2^2$.

Then:

$$\left(\frac{1}{x}\right)^k = \prod_{i: b_i = 1} \left(\frac{1}{x}\right)^{2^i} \tag{S19}$$

Computing Powers of 2

We compute iteratively:

$$\begin{aligned}
\left(\frac{1}{x}\right)^1 &= \sum_S a_S \prod_{j\in S} q_j \\
\left(\frac{1}{x}\right)^2 &= \left[\left(\frac{1}{x}\right)^1\right]^2 \\
\left(\frac{1}{x}\right)^4 &= \left[\left(\frac{1}{x}\right)^2\right]^2 \\
\left(\frac{1}{x}\right)^8 &= \left[\left(\frac{1}{x}\right)^4\right]^2 \\
&\vdots
\end{aligned} \tag{S20}$$

Coefficient Recurrence

When squaring $(1/x)^{2^i}$ to get $(1/x)^{2^{i+1}}$:

If $(1/x)^{2^i} = \sum_T c_T \prod_{j\in T} q_j$, then:

$$(1/x)^{2^{i+1}} = \sum_T d_T \prod_{j\in T} q_j \tag{S21}$$

where:

$$d_T = \sum_{\substack{S_1,S_2\subseteq\{0,1,\ldots,n-1\} \\ S_1\cup S_2=T}} c_{S_1} \cdot c_{S_2} \tag{S22}$$

Example: Computing $(1/x)^{12}$

Decomposition: $12 = 8 + 4 = 2^3 + 2^2$

Steps:

1. Compute coefficients a_S for $(1/x)^1$
2. Square to get coefficients for $(1/x)^2$

3. Square to get coefficients for $(1/x)^4$
4. Square $(1/x)^4$ to get coefficients for $(1/x)^8$
5. Multiply $(1/x)^8 \cdot (1/x)^4$ to get $(1/x)^{12}$

Note that for some collision systems often involving non-s states, odd inverse powers exist in potential energy surface fits (e.g. x^{-3}, x^{-11}). In these instances, odd inverse powers can be obtained by multiplying the appropriate intermediate terms, which exist on separate arithmetic registers (e.g. $x^{-1} \times x^{-2} = x^{-3}$ requires three separate arithmetic registers).

For the final multiplication:

$$b_T^{(12)} = \sum_{\substack{S_1, S_2 \subseteq \{0,1,\ldots,n-1\} \\ S_1 \cup S_2 = T}} b_{S_1}^{(8)} \cdot b_{S_2}^{(4)} \tag{S23}$$

For a set T, there are $2^{|T|}$ subsets, $3^{|T|} - 2^{|T|+1} + 1$ ordered pairs (S_1, S_2) with $S_1 \cup S_2 = T$, with the number of ordered k-tuples $(S_1, \ldots S_k)$ with $\bigcup_{i=1}^{k} S_i = T$ given by the inclusion-exclusion formula.

Complexity Analysis

The inverse polynomial circuit requires $\mathcal{O}(2^n - 1)$ multi-controlled R_z gates prior to decomposition into native two-qubit gates, reducing to $\mathcal{O}(2^{n-1})$ layers after CNOT cascade decomposition. The function $f(r) = 1/r$ evaluated on a linearly spaced binary grid of $N = 2^n$ points has no known symmetry in the multilinear basis: all 2^n coefficients a_S are nonzero and distinct, requiring $\mathcal{O}(2^n)$ independent rotation angles specifying $\mathcal{O}(2^n)$ independent function values. This constitutes an information-theoretic bound, wherein loading a linearly-spaced coordinate as a function value inherently requires $\mathcal{O}(2^n)$ independent parameters.

Exponential scaling is in simulation box size (n qubits), not in the physical dimensionality of the problem. In a multidimensional or multi-channel extension, the inverse polynomial

circuit replaces a harmonic function in the on-diagonal block, retaining polynomial complexity in the number of dimensions and channels. Further, $n \propto \log(L\sqrt{E_{char}})$. While we retain the perspective of gate count scaling exponentially with register, computational chemists and quantum algorithms researchers may instead state the complexity more favorably, viewing scaling as being linear in box size and sublinear in characteristic energy. Both perspectives are valuable in the present context, though we believe the register-based perspective is more informative to the practical limitations of simulating molecular dynamics.

In this finite limit, the maximum energy the potential energy surface can take for a function $V(x) = \left(\frac{1}{x}\right)^k$ is $\left(\frac{1}{\Delta x}\right)^k$, where Δx is the width of the spatial discretization on axis x. If the position register is given by $n = 10$ qubits for a box size $L = 40$, we find $\Delta x = L/(2^n) = 40/(2^{10}) = 5/128$. For a Coulomb potential, the maximum potential energy becomes 25.6 Hartrees (staying in atomic units), and $\approx 7.92 \times 10^{16}$ Hartrees for a Lennard-Jones potential. The potential can be horizontally shifted (regularized) at no extra cost to commensurately lower its maximum energy, and by extension the spectral norm of the operator.

Discretization of the position grid Δx scales as 2^{-n} for n qubits in the position basis. A simulation box size L is chosen to fit all desired dynamics both in position space and in momentum space. Momentum space is given by the initial momentum plus or minus the Nyquist frequency, given by $\mathcal{N} = \pi/(\Delta x)$. The maximum resolvable kinetic energy is therefore $\mathcal{N}^2/2\mu$.

For a constant L, the momentum simulation box doubles in size for every additional qubit added to the position register. Momenta not captured within the Nyquist range will appear on the opposite side of the range due to periodic boundary conditions and impact system dynamics. High energy eigenstates therefore require additional qubits for an acceptable Nyquist range.

For the direct computation of $(1/x)^k$, the complexity scales with the number of k-tuples $(S_1, ..., S_k)$ with fixed union T as $O(2^{k|T|})$, leading to a total complexity of $O(k \cdot 2^{kn})$. The

number of squaring operations scales as $O(\log k)$, and each squaring requires $O(4^n)$ operations, leading to a total complexity $O(4^n \log k)$.

The degree of parallelism that can be achieved in the classical precomputation and caching of these rotational parameters is substantial, with each k-tuple capable of being calculated independently, and further subdivided by fixed unions therein to maximize for the parallelism available, requiring a simple summing procedure after calculation. So, despite being an exponentially complex classical calculation, the exponential parallelism affords cooperation with existing high performance computing frameworks. Further, this calculation is a one-time precomputation, reducing overall computational load for all simulations using these potential surfaces.

Numerical Precision of Rotation Angles

We can assess the numerical precision required by examining the outputs for given register sizes. For example, coefficients for the following subsets on $n = 15$ qubits for a polynomial $f(x) \propto \frac{1}{x^{12}}$ are given below:

```
{0}: 1.0000000000000000e+0
{1}: 2.4414062500000000e-4
{2}: 5.9604644775390630e-8
{3}: 1.4551915228366852e-11
{4}: 3.5527136788005010e-15
{5}: 8.6736173798840350e-19
{6}: 2.1175823681357510e-22
{7}: 5.1698788284564230e-26
{8}: 1.2621774483536190e-29
{9}: 3.0814879110195774e-33
{10}: 7.5231638452626400e-37
{11}: 1.8367099231598242e-40
{12}: 4.4841550858394150e-44
{13}: 1.0947644252537633e-47
{14}: 2.6727647100921956e-51
```

As the trivial case, the first layer of single-qubit rotations follow the relation $\phi_{\{i\}} = \left(2^{-k}\right)^i$ for an inverse polynomial of degree k. Coefficients are given as arithmetic combinations, so factors on the order of 1 are often summed with factors on the order of $\left(2^{-k}\right)^n$. These were chosen as a small sample to show the degree of precision necessary. While bitwise data storage can handle these single-qubit numbers, long double precision is found to be insufficient for other rotation angles. For these coefficients, we truncated the value to long-double precision with no special handling of additional significant figures. This unfortunately presents itself as the number of T-gates (referring to the quantum gate, not the union of subsets as previously defined) scaling linearly with register size. The impact of reducing numerical precision and the relation to state fidelity is not explicitly considered here. We note that removing all gates whose base coefficients give rotation angles below any reasonable threshold does not reduce the complexity of the circuit, with half of all gates having rotation angles with magnitudes

greater than 1, and additional precision being required for terms in the sum.

Considering modern quantum hardware as of the date of publication, rotation angles of the magnitude of even 10^{-4} prove difficult, with recent publications for trapped ion quantum computers obtaining precision of $\pi/500 \approx 6.283 \times 10^{-3}$. Current coherence times further leave this work in the realm of classical emulation and heuristic, with further optimizations for Trotterized quantum circuits and exponentially-scaling potential energy surfaces required for even fault-tolerant applicability.

It can be noticed from the analytical form, but it bears mentioning that all coefficients calculated for a subset of qubits S_n for a quantum register of n qubits are the same as the coefficients calculated for a subset of qubits S_m for a quantum register of m qubits if $S_n = S_m$. An optimal $n = 15$-qubit calculation will therefore only need to calculate the coefficients for subsets containing the last qubit, since the smaller registers calculated the coefficients for all of the other subsets for that polynomial degree. The library provided in the Data Availability section of the main text gives coefficients up to long double precision for $n = 3 - 15$ qubits and polynomial degrees $k = 1 - 16$. The C-integrated Python script to calculate these coefficients does not take advantage of this fact.

Extension to Multi-Channel Scattering

Perhaps the largest demand of quantum computers for quantum scattering is the efficient handling of a multi-channel extension of a scattering problem. Any classical eigenstate precomputation remains exponentially complex. Recent research handling quantum scattering problems have focused on the extraction of phase shifts after a time evolution constructed using the eigenstate.[1] This reduces the number of measurements required to extract a phase shift accurately, though these methods do not explicitly consider the computational bottleneck of the scattering problem. This section describes the handling of multiple quantum channels and potential energy surfaces.

An ancilla register can be used to control the activation of terms dictating system dynamics.[2–4] In chemical dynamics, these ancillary qubits store the relative population in each potential energy surface in superposition, activating the potentials according to their population by controlled operations. In the instance of quantum scattering, the populations for each quantum channel can therefore be stored in $\lceil \log_2 M \rceil$ for M potential surfaces. Each surface is applied independently, increasing depth by a factor linear in M.

Potential couplings can be defined globally, or by position-dependent functions. Ollitrault et al. (2020)[2] implement linear piecewise couplings between nonadiabatic potential energy surfaces using two additional registers, the first as a computational register and the second to store truth values for the position of a wavepacket relative to reference basis states. The result is an algorithm with a log-scaled depth and linear-scaled register size, given by (for example) ripple-carry addition circuits.[5,6] A $\mathcal{O}(P-1)$-size register stores truth values associated with wavepacket position, to apply a piecewise-linear approximation of a potential coupling with P-pieces. More information on the application of coupling terms can be found in the supporting information of Ollitrault et al. (2020).[2]

For $M > 1$ electronic channels, each surface $V_{ii}(r)$ carries its own interaction parameters and partial-wave quantum numbers. The multilinear coefficients are generated independently for each surface and assembled into uniformly controlled rotation (UCR) angle vectors with $m = \lceil \log_2 M \rceil$ channel register qubits as controls. Gate cost per rotation site increases from one unconditional R_z to M rotations interleaved with $M-1$ CNOTs, but the number of rotation sites and the gate count invariance under polynomial power remain unchanged. Precomputed coefficient libraries can be reused across channels by rescaling with surface-dependent prefactors. The total on-diagonal depth is $\mathcal{O}(M \cdot 2^n)$ per Trotter step, reducible to $\mathcal{O}(Mn^2)$ through quantum multiplication (arithmetic), with efficient approximability guaranteed by syllogism from the Chang et al. proof for the BRGC encoding.[7] Off-diagonal depth is $\mathcal{O}(M^2 P)$ for piecewise-linear couplings with P pieces, rising to $\mathcal{O}(M^2 \cdot 2^n)$ for arbitrary smooth coupling functions.[4] Extension to multi-mode scattering ($d > 1$) places each spatial

or internal mode on its own n-qubit register, with the inverse polynomial circuit providing the intra-mode potential, inter-mode bilinear terms handled by the on-diagonal protocol, and fan-out protocols adding $(M-1)(d-1)$ ancilla qubits for parallel execution.

Effective Rotation Angle Assembly

Each term in the effective potential $V_{\mathrm{eff}}(r) = V_{\mathrm{LJ}}(r) + V_{\mathrm{cf}}(r)$ expands independently on the qubit basis using the repeated squaring algorithm: the repulsive wall $(\sigma/r)^{12} \propto r^{-12}$ uses coefficients $b_T^{(12)}$ with prefactor $4\epsilon\sigma^{12}$; the attractive well $-(\sigma/r)^6 \propto r^{-6}$ uses coefficients $b_T^{(6)}$ with prefactor $-4\epsilon\sigma^6$; the centrifugal barrier $\propto r^{-2}$ uses coefficients $b_T^{(2)}$ with prefactor $\ell(\ell+1)/(2\mu)$.

For each subset T, values are summed into a single effective rotation angle,

$$\theta_T = 2\pi\tau \left[4\epsilon\,\sigma^{12}\,b_T^{(12)} - 4\epsilon\,\sigma^6\,b_T^{(6)} + \frac{\ell(\ell+1)}{2\mu}\,b_T^{(2)}\right], \tag{S24}$$

and the full effective potential circuit is

$$e^{-iV_{\mathrm{eff}}\tau} = \prod_{T\subseteq\{0,1,\ldots,n-1\}} C^{|T|}R_z(\theta_T), \tag{S25}$$

using $|T|$-controlled R_z rotations, decomposed by UCR into CNOT ladders and $\mathcal{O}(2^n-1)$ uncontrolled R_z rotations.[8]

Classical precomputation of these coefficients depends only on n, Δ, and the power α. Once computed, the coefficients can be cached and reused for all simulations with different physical parameters (ϵ, σ, ℓ, μ), since the physical prefactors multiply the cached values at assembly time.

Operator Validation: Hilbert–Schmidt Distance

The Hilbert–Schmidt distance between the binary-encoded potential circuit and the independently derived binary-reflected Gray code (BRGC) construction of Chang et al. (2022)[7] was computed for r^2, $1/r$, and the full Lennard–Jones potential. The two unitary matrices agree to numerical precision, with Hilbert–Schmidt distance vanishing to the level of double-precision arithmetic ($\sim 10^{-15}$) for the Harmonic and Coulomb potentials, validating the circuit construction without reliance on any classical approximation or scattering-specific observable.

For the full Lennard–Jones potential, coefficients scale $\propto (2^n)^{12-1}$ under repeated squaring (Equation S.12 with $\alpha_{S_\alpha} \propto 2^n$). When computed to 64-bit precision, repeated squaring coherently accumulates machine precision error, increasing the distance between each circuit construction and between circuits and the target potential shape (Figure S1a). This leads to extreme differences for an unregularized Lennard–Jones potential, with unweighted parameters increasing as $(2^n)^{12-1}$ and accumulating significant error, impacting operator accuracy near the repulsive boundary. Coulomb and Harmonic potentials follow a consistent error accumulation, with exponential scaling in n following from the exponential number of double-precision arithmetic operations with coherent error accumulation.

This effect is suppressed under regularization, where the Lennard–Jones potential is shifted to the characteristic energy of the incoming particle. The resulting circuit is also closed-form on the interval $[0, L]$ and retains improved numerical stability.

Parameter magnitudes for the Coulomb potential on $n = 10$ qubits for the binary and BRGC methods are given in Figures S1b and S1c, where magnitude distributions become inverted and rescaled. As confirmed numerically, BRGC coefficients blow up near the hard-core singularity, a trait shared with the binary encoding.[7] While these reduce to values between 0 and 2π in practice, large precise values need special handling in classical precomputation to store far more decimal places than double-precision affords. This sensitivity to parameter magnitude suggests that, prior to the mod 2π operation to convert the parameter to a

rotation angle, parameters need additional precision in classical precomputation.

Binary multilinear decomposition and the BRGC Walsh–Hadamard decomposition produce operators identical up to numerical precision, validating the circuit construction without reliance on any classical approximation or scattering-specific observable.

Quantum Arithmetic Alternative

An alternative to the multilinear expansion and BRGC protocol is to compute $1/r^{\alpha}$ via quantum arithmetic circuits acting on ancilla registers, reducing gate count from exponential to polynomial in n at the cost of additional qubits and Toffoli gates. This section analyzes the overhead of such an approach for the Lennard–Jones $1/r^{12}$ potential used in the He–He scattering simulation, comparing against the UCR-decomposed multilinear scheme.

Arithmetic Pipeline

The pipeline transforms the binary-encoded position register $|r\rangle$ into a fixed-point representation of $1/r^{12}$ via five reversible arithmetic operations, applies diagonal phase rotations on the result register, then uncomputes to restore all ancilla qubits to $|0\rangle$:

1. **Reciprocal:** $|r\rangle|0^{n_a}\rangle \to |r\rangle|s_1 = 1/r\rangle$ into an n_a-bit ancilla register.
2. **Square (1):** $|s_1\rangle|0^{n_a}\rangle \to |s_1\rangle|s_2 = 1/r^2\rangle$.
3. **Square (2):** $|s_2\rangle|0^{n_a}\rangle \to |s_2\rangle|s_3 = 1/r^4\rangle$.
4. **Square (3):** $|s_3\rangle|0^{n_a}\rangle \to |s_3\rangle|s_4 = 1/r^8\rangle$.
5. **Multiply:** $|s_3\rangle|s_4\rangle|0^{n_a}\rangle \to |s_3\rangle|s_4\rangle|s_5 = 1/r^{12}\rangle$.

The phase $e^{-i\alpha\tau\cdot s_5}$ is then applied as n_a single-qubit R_z rotations on the output register:

$$e^{-i\alpha\tau\, s_5} = \prod_{\ell=0}^{n_a-1} R_z\big(2\alpha\tau\,\Delta_a\,2^{\ell}\big)\,, \tag{S26}$$

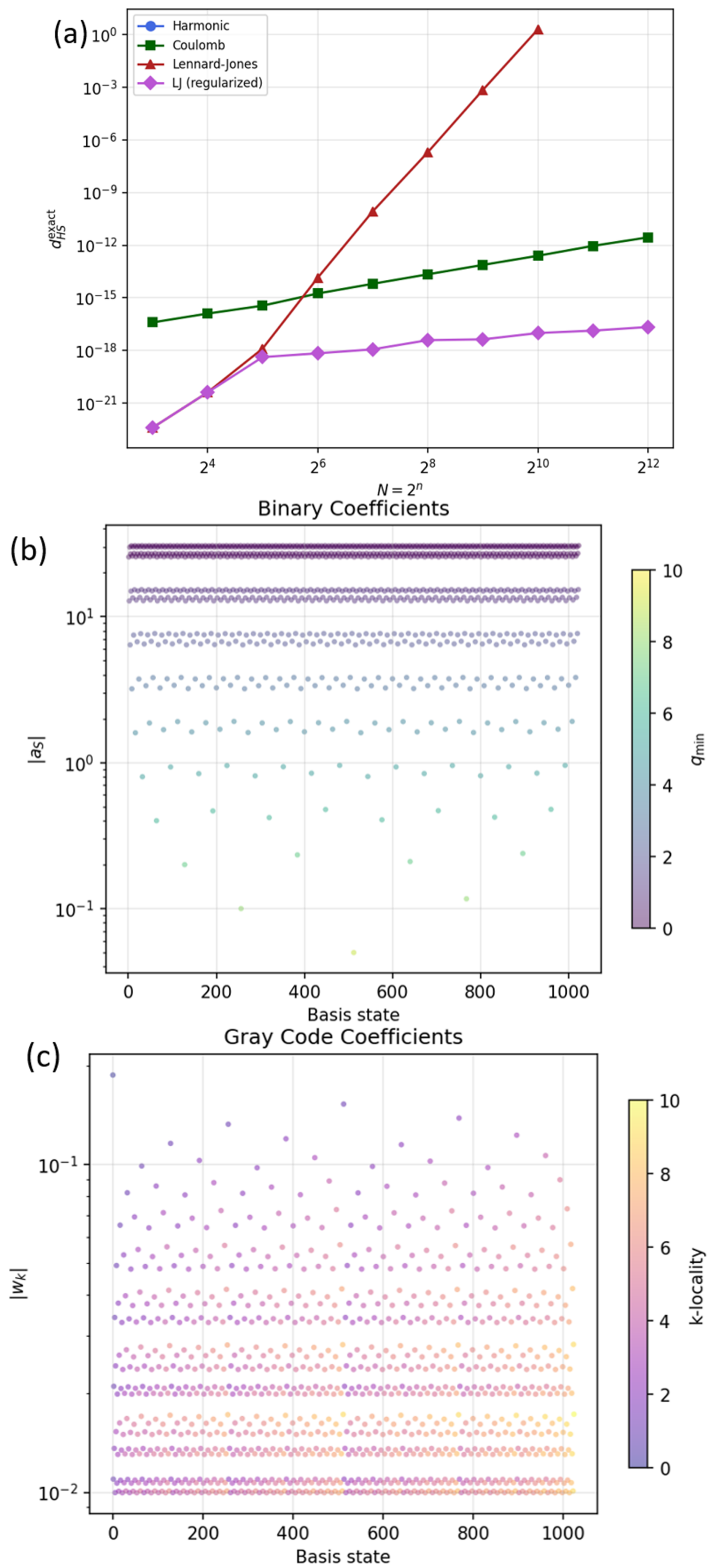

Figure S1: a) Hilbert–Schmidt distance between the binary-encoded circuit and the BRGC circuit for varying register sizes $N = 2^n$. Harmonic and Coulomb potentials maintain distances near machine floor (double precision) ($\sim 10^{-15}$), while the unregularized Lennard–Jones potential diverges due to coherent error accumulation under repeated squaring. The Hilbert-Schmidt distance for the Harmonic term directly overlaps with the 'LJ (regularized)' points. b) Binary coefficients $|a_S|$ for the Coulomb potential on $n = 10$ qubits. Color gradient denotes the number of control qubits prior to UCR decomposition. c) Walsh coefficients $|w_k|$ for the BRGC potential on $n = 10$ qubits. Color gradient denotes the k-locality of the associated operation.

where Δ_a is the least-significant-bit weight of the fixed-point representation.

Ancilla Precision Requirement

The smallest nonzero position on the binary grid is $r_{\min} = \Delta = L/2^n$, giving a maximum inverse twelfth power of $(2^n/L)^{12}$. The maximum phase angle is

$$\theta_{\max} = |\alpha\tau| \cdot (2^n/L)^{12}, \tag{S27}$$

where $\alpha = 4\epsilon\sigma^{12}$ for the repulsive Lennard–Jones term. The number of ancilla bits required to resolve $\theta \bmod 2\pi$ to precision ε_θ is

$$n_a = \left\lceil \log_2\left(\frac{\theta_{\max}}{\varepsilon_\theta}\right)\right\rceil. \tag{S28}$$

For the He–He system ($n = 13$, $L = 200\,\text{a.u.}$, $\epsilon = 3.236 \times 10^{-5}\,\text{E}_h$, $\sigma = 4.831\,\text{a.u.}$, $\tau = 1\,\text{a.u.}$): $\alpha\tau \approx 2.09 \times 10^4\,\text{a.u.}$, $(2^{13}/200)^{12} \approx 2.23 \times 10^{19}$, giving $\theta_{\max} \approx 4.67 \times 10^{23}$. With per-gate synthesis precision $\varepsilon_\theta = 10^{-10}$:

$$n_a = \left\lceil \log_2(4.67 \times 10^{33})\right\rceil = 112. \tag{S29}$$

The arithmetic register therefore exceeds the position register by nearly an order of magnitude ($n_a/n \approx 8.6$).

Toffoli Gate Counts

The forward-pass Toffoli count for the $1/r^{12}$ pipeline is

$$T_{\text{fwd}} = \underbrace{4n_a^2}_{\text{Reciprocal}} + \underbrace{3\,n_a(n_a+1)}_{\text{3 squarings}} + \underbrace{4n_a^2}_{\text{Multiply}} = 11n_a^2 + 3n_a. \tag{S30}$$

Table S1: Toffoli gate counts for the reversible arithmetic primitives used in the $1/r^{12}$ pipeline.

Operation	Toffoli count	Notes
Multiplier ($n_a \times n_a$)	$2n_a^2$	incl. carry propagation
Squarer ($n_a \times n_a$)	$n_a(n_a-1)/2 + n_a$	truncated via Bennett's trick
Reciprocal (Newton, p iter.)	$\leq 4n_a^2$	$p = \lceil \log_2 n_a \rceil$ iterations

Including the reverse pass (uncomputation):

$$T_{\text{arith}} = 2\,T_{\text{fwd}} = 22\,n_a^2 + 6\,n_a. \tag{S31}$$

T-Gate Cost Comparison

Each Toffoli decomposes into 4 T-gates; each R_z rotation is synthesized at 23 T-gates.[9,10]

The per-application T-gate counts are:

$$T^{(\text{UCR})} = 23\,(2^n - 1), \qquad T^{(\text{arith})} = 88\,n_a^2 + 47\,n_a. \tag{S32}$$

For He–He ($n = 13$, $n_a = 113$): $T^{(\text{UCR})} = 1.88 \times 10^5$ and $T^{(\text{arith})} = 1.13 \times 10^6$, a ratio of $6.0\times$ in favor of the UCR scheme.

The T-depth comparison yields: $D^{(T)}_{\text{UCR}} = 1.88 \times 10^5$ and $D^{(T)}_{\text{arith}} = 32\,n_a(p+5/2) + 23\,n_a = 2.76 \times 10^5$ ($p = 7$), a ratio of $1.5\times$ in favor of UCR.

The total ancilla overhead for the arithmetic scheme is approximately $6\,n_a = 678$ qubits.

Crossover Analysis

A T-gate crossover occurs when $T^{(\text{arith})} = T^{(\text{UCR})}$:

$$88\,n_a^2 + 47\,n_a = 23\,(2^n - 1). \tag{S33}$$

For the Lennard–Jones potential with parameters comparable to the He–He system, taking $n_a \approx 9n$ as representative: $88(9n)^2 \approx 7{,}128\, n^2$, and the crossover $7{,}128\, n^2 = 23 \times 2^n$ gives $n^\star \approx 15$. For smaller systems ($n < 15$), the UCR scheme dominates. Even at the crossover, the arithmetic scheme requires hundreds of ancilla qubits, a cost exacerbated in the multi-channel, multi-mode extension.

Partial-Wave Truncation

We truncate the partial wave sum at $\ell_{\max} = 18$. The radial equation is evaluated for $\ell = 30$ at $k_0 = 0.2173\, a_0^{-1}$, giving $\delta_{18} = 3.7 \times 10^{-6}$ rad and $\delta_{22} = 1.4 \times 10^{-6}$ rad. The integral cross section accumulates a resulting truncation error given below.

$\ell_{\max}$	σ_{bos} (a_0^2)	relative error
10	80.3757	1.6×10^{-7}
12	80.3757	4.6×10^{-8}
15	80.3757	1.5×10^{-8}
18	80.3757	2.4×10^{-9}
30	80.3757	—

As both the time-dependent quantum circuit compilation and the time-independent methods both truncate at the same $\ell_{\max}$, truncation is determined to be a negligible source of uncertainty between computations.

Momentum Resolution and the s-Wave

We bound phase-shift error by Equation 13 in the main text, with phase-shift error arising from the finite momentum grid whose bounds are defined by the box size and grid resolution after a quantum Fourier transform, as $\varepsilon_\delta \lesssim |d\delta_\ell/dk|\, \pi/L$. We give the time-independent solution's derivative at the initial momentum $k_0 = 0.2173\, a_0^{-1}$ below:

The s-wave phase shift is more sensitive to grid spacing $\Delta_p = 2\pi\hbar/L$, deviating by 34% of the bound, as do $\ell = 2$ and $\ell = 3$. Meanwhile, $\ell = 1$ has a tighter bound not accounted for

ℓ	0	1	2	3
$d\delta_\ell/dk$ (a_0)	-5.74	$+0.13$	$+2.41$	$+0.45$
bound at $L = 200\,a_0$ (rad)	0.090	0.0021	0.038	0.0071
observed $\lvert\Delta\delta_\ell\rvert$ (rad)	0.0311	0.0222	0.00024	0.0019

by Nyquist limits, and the value exceeding this bound we instead report as a residual. The bound decreases as $1/L$, so a box that is four times larger (two extra qubits at the same grid resolution) reduces s-wave contribution to the symmetrized cross-section result. The discrepancy lies below one percent at fixed ε_k.

References

(1) Turro, F.; Wendt, K. A.; Quaglioni, S.; Pederiva, F.; Roggero, A. Evaluation of phase shifts for nonrelativistic elastic scattering using quantum computers. *Physical Review C* **2024**, *110*, 054604.

(2) Ollitrault, P. J.; Mazzola, G.; Tavernelli, I. Nonadiabatic molecular quantum dynamics with quantum computers. *Physical Review Letters* **2020**, *125*, 260511.

(3) Chawdhry, H. A.; Pellen, M.; Williams, S. Quantum simulation of scattering amplitudes and interferences in perturbative QCD. *arXiv preprint arXiv:2507.07194* **2025**,

(4) Courtney, J. An oracle-free quantum algorithm for nonadiabatic quantum molecular dynamics. *arXiv preprint arXiv:2604.19319* **2026**,

(5) Cuccaro, S. A.; Draper, T. G.; Kutin, S. A.; Moulton, D. P. A new quantum ripple-carry addition circuit. *arXiv preprint quant-ph/0410184* **2004**,

(6) Draper, T. G.; Kutin, S. A.; Rains, E. M.; Svore, K. M. A logarithmic-depth quantum carry-lookahead adder. *arXiv preprint quant-ph/0406142* **2004**,

(7) Chang, C. C.; McElvain, K. S.; Rrapaj, E.; Wu, Y. Improving schrödinger equation im-

plementations with gray code for adiabatic quantum computers. *PRX Quantum* **2022**, *3*, 020356.

(8) Möttönen, M.; Vartiainen, J. J.; Bergholm, V.; Salomaa, M. M. Quantum circuits for general multiqubit gates. *Physical review letters* **2004**, *93*, 130502.

(9) Ross, N. J.; Selinger, P. Optimal ancilla-free Clifford+ T approximation of z-rotations. *Quantum Inf. Comput.* **2016**, *16*, 901–953.

(10) Gidney, C. Halving the cost of quantum addition. *Quantum* **2018**, *2*, 74.
